# Sex hormone-binding globulin exerts sex-related causal effects on lower extremity varicose veins: evidence from gender-stratified Mendelian randomization

**DOI:** 10.3389/fendo.2023.1230955

**Published:** 2023-12-11

**Authors:** Qinglu Fan, Yang Meng, Zhihao Nie, Songping Xie, Changzheng Chen

**Affiliations:** ^1^ Department of Thoracic Surgery, Renmin Hospital of Wuhan University, Wuhan, China; ^2^ Department of Ophthalmology, Renmin Hospital of Wuhan University, Wuhan, China

**Keywords:** sex hormone, sex hormone-binding globulin, sex difference, lower extremity varicose veins, gender-stratified Mendelian randomization

## Abstract

**Background:**

The association between serum sex hormones and lower extremity varicose veins has been reported in observational studies. However, it is unclear whether the association reflects a causal relationship. Besides, serum sex hormone-binding globulin (SHBG) has been rarely studied in lower extremity varicose veins. Here, we aim to investigate the association between serum levels of SHBG, testosterone, and estradiol and the risk of lower extremity varicose veins using Mendelian randomization (MR).

**Methods:**

We obtained genome-wide association study summary statistics for serum SHBG levels with 369,002 European participants, serum testosterone levels with 424,907 European participants, serum estradiol levels with 361,194 European participants, and lower extremity varicose veins with 207,055 European participants. First, a univariable MR was performed to identify the causality from SHBG and sex hormone levels to lower extremity varicose veins with several sensitivity analyses being performed. Then, a multivariable MR (MVMR) was performed to further assess whether the causal effects were independent. Finally, we performed a gender-stratified MR to understand the role of genders on lower extremity varicose veins.

**Results:**

Genetically predicted higher serum SHBG levels significantly increased the risk of lower extremity varicose veins in the univariable MR analysis (OR=1.39; 95% CI: 1.13–1.70; P=1.58×10^-3^). Sensitivity analyses and MVMR (OR=1.50; 95% CI:1.13-1.99; P=5.61×10^-3^) verified the robustness of the causal relationships. Gender-stratified MR revealed that higher serum SHBG levels were associated with lower extremity varicose veins in both sexes. However, the OR of serum SHBG levels on lower extremity varicose veins risk in females (OR=1.51; 95% CI: 1.23–1.87; P=1.00×10^-4^) was greater than in males (OR=1.26; 95% CI: 1.04–1.54; P=1.86×10^-2^).

**Conclusions:**

Serum SHBG levels are positively related to lower extremity varicose veins risk in both sexes, especially in females. This may partly explain the higher prevalence of varicose vines among females.

## 1 Introduction

Lower extremity varicose veins are engorged and dilated veins that happen when incompetent or damaged valves cause bidirectional or reverse blood flow in the deep veins, perforator veins, and great and/or small saphenous veins (1, 2). As a major kind of chronic venous disease, lower extremity varicose veins are very common, affecting approximately 23% of the US adult population (3). Currently, 22 million women and 11 million men aged 40-80 years in the US are suffering from this condition (3, 4). The presence of lower extremity varicose veins not only impairs patients’ quality of life, but also increases the risk of venous complications, such as deep vein thrombosis, thrombophlebitis, skin changes, and chronic venous ulceration (4, 5). Moreover, the high prevalence of lower extremity varicose veins places a heavy burden on the healthcare system, mostly due to advanced cases with complications (6). Thus, it remains important to identify potential risk factors to facilitate early diagnosis and timely intervention for lower extremity varicose veins.

Sex hormones, including androgens and estrogens, play a vital role in the normal growth and development of the body. Traditional observational studies have suggested that serum sex hormone levels are linked to a variety of diseases, such as rheumatoid arthritis (7), atherosclerosis (8), stroke (9), chronic kidney disease (10), Alzheimer’s disease, Parkinson’s diseases (11), and lower extremity varicose veins (12, 13). Specifically, Ciardullo et al. found that high endogenous estradiol levels are associated with increased venous distensibility and clinical evidence of lower extremity varicose veins in menopausal women (14). Moreover, it has been noticed that elevated serum estradiol/free testosterone ratio is not only associated with lower extremity varicose veins in males (15), but a predictor for recurrent lower extremity varicose veins in those who underwent surgical treatment (16).

Nevertheless, limited attention has been paid to the association between serum sex hormone-binding globulin (SHBG), a circulating glycoprotein functioning as a transporter of sex hormones, and lower extremity varicose veins (17). Moreover, traditional studies, due to their inherent defects, have limited ability to preclude reverse causality and confounding factors, both of which may hinder causal inference between SHBG and lower extremity varicose veins (18).

Mendelian randomization (MR) is a research method for causal inference based on the random assortment of genetic variants during meiosis (the law of independent assortment) (19). MR utilizes exposure-related genetic variations as instrumental variables (IVs) to assess whether the association between the exposure and outcome is consistent with a causal effect (19). Since genetic variants are randomly assigned at conception prior to disease onset, MR analysis could efficiently identify causal determinants of a certain outcome and exclude reverse causality and confounding factors (20).

In this study, we conducted an MR analysis using large-scale genome-wide association study (GWAS) summary data to investigate the relationships between the serum levels of SHBG, testosterone, and estradiol (as exposures) and the risk of lower extremity varicose veins (as outcome).

## 2 Materials and methods

### 2.1 Study design

**Figure 1:**
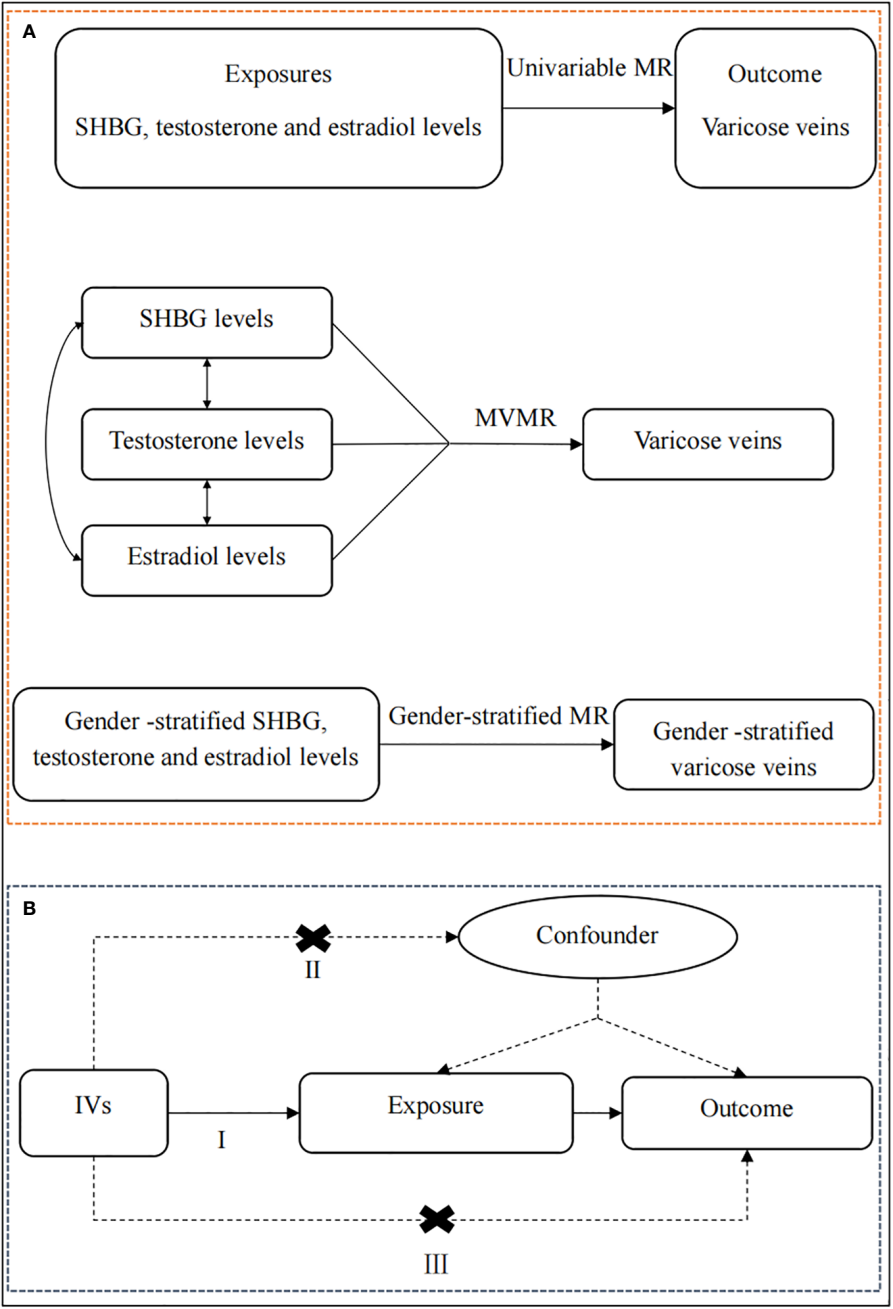
The overview of the study design. **(A)** Flow chart of the univariable MR, MVMR, and gender-stratified MR. First of all, we performed a univariable MR analysis to explore whether genetically predicted serum levels of SHBG, testosterone, and estradiol (exposures) were associated with the risk of lower extremity varicose veins (outcome). Then, for exposures that survived the univariable MR, we conducted a multivariable MR analysis to estimate the independent causal effect of these exposures on lower extremity varicose veins. Finally, for exposures that survived the above screening criteria, we further perform a gender-stratified MR analysis to understand the roles of genders on lower extremity varicose veins using gender-stratified GWAS datasets. **(B)** The schematic chart of the three basic principles of MR: (I) IVs are associated with the exposure; (II) IVs are independent of potential confounders; (III) IVs are associated with the outcome through the studied exposure only. SHBG, sex hormone-binding globulin; MR, Mendelian randomization; MVMR, multivariable Mendelian randomization; IVs, instrumental variables.

Since this study used previously collected and publicly available GWAS data, no additional ethical approval was required. The overview of the study design is presented in **Figure 1**.

### 2.2 Data source

Summary data for serum levels of SHBG (females and males) and testosterone (females and males) were obtained from the published GWAS dataset including over 400,000 European participants with the genotyping chip, age at baseline, and first ten genetically derived principal components as covariates (21). Specifically, 369,002 European participants were included in serum SHBG levels and 424,907 European participants in serum testosterone levels. For SHBG, body mass index was used as a complementary covariate which has been previously demonstrated to increase statistical power by reducing trait variance (21). Detailed information about participants in SHBG and testosterone was shown in **Supplementary Table S1**. In addition, summary data for serum estradiol levels (females and males) was obtained from the IEU OPEN GWAS project (https://gwas.mrcieu.ac.uk/; ID ukb-d-30800_raw) with 361,194 European participants. Detailed information about participants in estradiol was shown in **Supplementary Table S1**.

Summary data for lower extremity varicose veins (females and males) was obtained from the latest published GWAS article including 207,055 participants of European ancestry (22). The cases were diagnosed with the international classification of diseases, tenth revision (ICD-10) code I83 or ICD-8/9 code 454. The data was adjusted for covariates including age, genotyping batch, ten principal components of ancestry, and the kinship matrix. Detailed information about participants in estradiol was shown in **Supplementary Table S1**.

### 2.3 Selection of instrumental variables

Only single-nucleotide polymorphisms (SNPs) that met the following screening criteria were selected as IVs: 1). SNPs with genome-wide significance threshold P < 5 × 10^-8^ were considered to be associated with exposures, thus seen as potential IVs; 2). SNPs were eliminated according to linkage disequilibrium (threshold r^2^=0.001, KB = 10000); 3). only SNPs with the F statistic ≥10 (F statistic =β_exposure_^2/SE_exposure_^2) were included, indicating no strong evidence of weak instrument bias (23, 24); 4). all palindromic SNPs were dropped; 5). the MR pleiotropy residual sum and outlier (MR-PRESSO) test was applied to detect potential horizontal pleiotropy and to eliminate the effects of pleiotropy by removing outlier SNPs (25).

### 2.4 MR analyses

We performed three MR analytical methods, including the random effects/fixed effects inverse variance weighted (RE/FE-IVW), weighted median (WM), and MR-Egger regression, to reveal the causal effect of exposures containing multiple IVs on lower extremity varicose veins. The Wald ratio method was used for the MR analysis of exposures containing only one IV. IVW was used as the main method whereas the MR-Egger and WM methods were used as supplements (26–28). IVW is calculated by regressing the coefficient from an outcome regression on the IV on that from an exposure regression on the variant and weighting each estimate by the inverse variance of the association between the instrument and the outcome (29). WM can provide consistent estimates when at least 50% of the weighted variances are from valid IVs (30). The MR-Egger regression method allows pleiotropy to present in more than 50% of IVs (29).

To verify the robustness of the identified causal associations, we carried out a series of sensitivity analyses, including Cochran’s Q test, the MR-Egger intercept test, and the leave-one-out analysis. The Cochran’s Q test was performed to estimate the heterogeneity among IVs associated with each exposure. The FE-IVW and RE-IVW were used when P > 0.05 and P < 0.05, respectively, to provide a more conservative but robust MR estimate (31). The MR-Egger intercept test was performed to detect the presence of horizontal pleiotropy (29). The leave-one-out analysis was performed to determine whether the significant results were driven by any single SNP (32).

In order to avoid the confounders among the serum SHBG, testosterone, and estradiol levels as much as possible, we conducted a multivariable MR (MVMR) analysis adjusted for each other. Finally, given that gender difference has been widely reported in lower extremity varicose veins, we further performed a gender-stratified MR analysis for significant associations that survived the above screening criteria, aiming to avoid potential sexual bias.

About statistical power, according to the methods described in lately published article by Hu et al. (33), the statistical power was calculated with an online tool (https://shiny.cnsgenomics.com/mRnd/) (34). The primary factors of statistical power are the sample size of the outcome and the proportion of variance in the exposure variable explained by the genetic instrument.

### 2.5 Statistics

First of all, we performed a univariable MR analysis to explore whether genetically predicted serum levels of SHBG, testosterone, and estradiol (exposures) were associated with the risk of lower extremity varicose veins (outcome). Then, for exposures that survived the univariable MR, we conducted a MVMR analysis to estimate the independent causal effect of these exposures on lower extremity varicose veins. Finally, for exposures that survived the above screening criteria, we further perform a gender-stratified MR analysis to understand the roles of genders on lower extremity varicose veins using gender-stratified GWAS datasets. Meanwhile, the MR study has to fulfill three assumptions: (I) IVs are associated with the exposure; (II) IVs are independent of potential confounders; (III) IVs are associated with the outcome through the studied exposure only (35).

All MR analyses were performed using the packages “TwoSampleMR” (version 0.5.6) and “MRPRESSO” (version 1.0) in R statistical software (version 4.2.3). The results of MR analyses were presented as odds ratios (OR) with 95% confidence intervals (CI) to quantify the association between exposures (serum SHBG, testosterone, and estradiol levels) and risk of lower extremity varicose veins. Due to multiple testing between exposures and lower extremity varicose veins, the MR analysis results to verify the causal effect were only considered statistically significant when Bonferroni corrected P < 0.017 (0.05/3) in the univariable MR and MVMR analyses while the threshold was set at P <0.05 in the gender-stratified MR analysis.

## 3 Results

### 3.1 Univariable MR analysis

In total, 492 SNPs were identified as IVs for serum SHBG, testosterone, and estradiol levels. Specifically, 356 SNPs were identified for SHBG levels, 141 SNPs for testosterone levels, and 1 SNP for estradiol levels, respectively. The F statistics of IVs ranged between 21.77 and 2,836.74, indicating no evidence of weak instrument bias. Detailed information on these IVs is listed in **Supplementary Table S2**.

**Figure 2:**
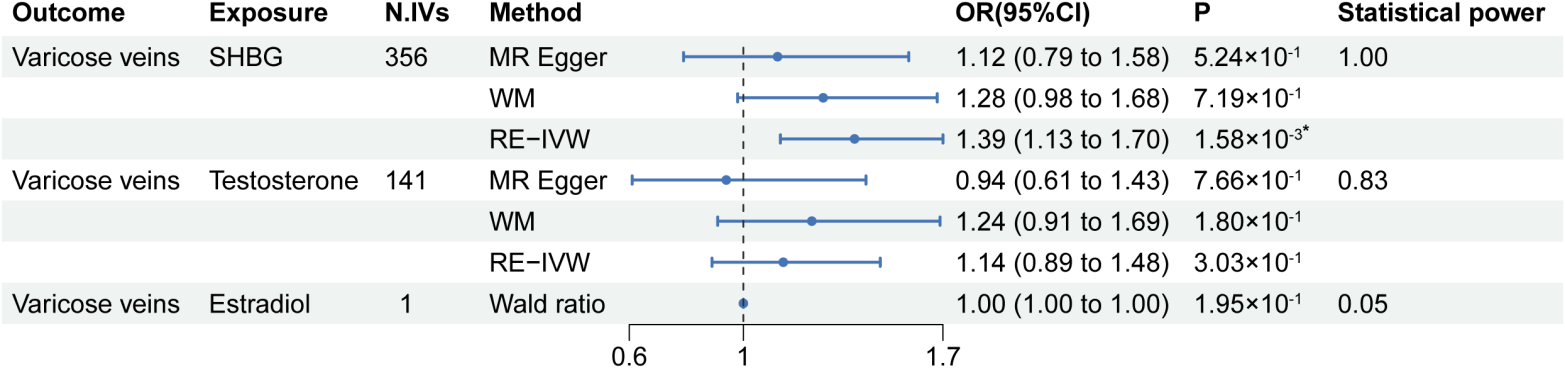
Results of the univariable MR analysis. MR, Mendelian randomization; SHBG, sex hormone-binding globulin; N. IVs, the number of IVs; WM, weighted median; RE-IVW, random-effects inverse variance weighted. *: P of statistical significance (<0.017) after Bonferroni correction.

The univariable MR results from serum SHBG, testosterone, and estradiol levels to lower extremity varicose veins are listed in **Figure 2**. Among the tested exposures, the MR estimates from the RE-IVW method indicated that higher serum SHBG levels significantly increased the risk of lower extremity varicose veins (OR=1.39; 95% CI: 1.13–1.70; P=1.58×10^-3^), and directions of the MR-Egger and WM methods were consistent with that of the IVW method. No evidence of causal associations between genetic liability for serum testosterone/estradiol levels and the risk of lower extremity varicose veins were shown. The scatter plots of IVs were shown in **Figure S1**. Meanwhile, the statistical power of serum SHBG levels, testosterone and estradiol were 1.00, 0.83 and 0.05, respectively.

The results of the Cochran’s Q test are shown in **Supplementary Table S3**. Heterogeneity was observed between the genetic IVs for SHBG and testosterone levels, thus the RE-IVW was used. Moreover, MR-Egger intercepts did not detect any pleiotropy, indicating no evidence of potential horizontal pleiotropy (all intercepts P > 0.05) (**Supplementary Table S3**). The leave-one-out analysis, as shown in **Supplementary Figure 2**, showed no marked difference in the causal effect of SHBG levels on lower extremity varicose veins, indicating that the significant results were not driven by any single SNP. No results for Cochran’s Q test and MR-Egger regression of estradiol levels were obtained due to insufficient IVs.

### 3.2 MVMR analysis

**Figure 3:**
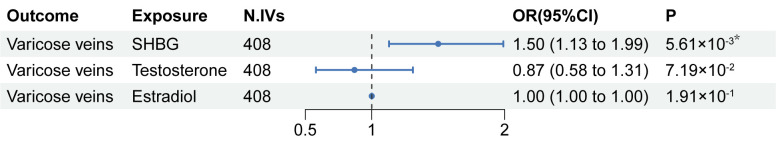
Results of the MVMR analysis. MVMR, multivariable Mendelian randomization; SHBG, sex hormone-binding globulin; N. IVs, the number of IVs. *: P of statistical significance (<0.017) after Bonferroni correction.

According to the results in the univariable MR analysis, serum SHBG levels seemed to be associated with lower extremity varicose veins. Nevertheless, since SHBG, testosterone, and estradiol were three interrelated substances, we further performed an MVMR analysis to avoid the confounders between serum SHBG, testosterone, and estradiol levels and to evaluate independent effects of the serum SHBG levels on lower extremity varicose veins. The results of the MVMR analysis are shown in **Figure 3**. In the MVMR analysis, higher serum SHBG levels remained causally related to a higher risk of lower extremity varicose veins. The OR of SHBG levels was 1.50 (95% CI:1.13-1.99; P=5.61×10^-3^), indicating that there was an independent causal effect of SHBG levels on lower extremity varicose veins. Moreover, the results of the remaining exposures (serum testosterone and estradiol levels) in the MVMR analysis were consistent with the previous univariable MR analysis.

### 3.3 Gender-stratified MR analysis

To further explore the causal-effect difference of genders on lower extremity varicose veins, a gender-stratified MR analysis was performed. In total, 411 SNPs were identified as IVs for gender-stratified serum SHBG levels, among which 236 SNPs were identified for SHBG in females, and 198 SNPs for males. The F statistics of IVs ranged between 20.62 and 1652.18, indicating no evidence of weak instrument bias. Detailed information on these IVs is listed in **Supplementary Table S4**.

**Figure 4:**
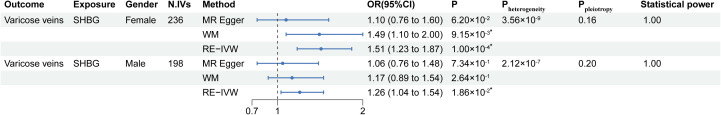
Results of the gender-stratified MR analysis. MR, Mendelian randomization; SHBG, sex hormone-binding globulin; N. IVs, the number of IVs. WM, weighted median; RE-IVW, random-effects inverse variance weighted. *: P of statistical significance (<0.005) after Bonferroni correction.

The MR results from gender-stratified serum SHBG levels to gender-stratified lower extremity varicose veins risk are listed in **Figure 4**. Gender-stratified MR showed that genetically predicted higher serum SHBG levels were associated with lower extremity varicose veins in both females and males, and the OR of serum SHBG levels on lower extremity varicose veins risk in females (OR_female_=1.51; 95% CI: 1.23–1.87; P=1.00×10^-4^) was greater than in males (OR_male_=1.26; 95% CI: 1.04–1.54; P=1.86×10^-2^). The scatter plots of IVs were shown in **Figure S3**. Moreover, the results from the Cochran’s Q and MR-Egger intercept tests are also shown in **Figure 4**. Heterogeneity was observed between the genetic IVs for SHBG levels in both females and males, for which the RE-IVW method was used. MR-Egger intercepts did not detect any pleiotropy, indicating no evidence of potential horizontal pleiotropy (both intercepts P > 0.05). Similar to the results of univariable MR analysis, the leave-one-out analysis showed that the significant results were not driven by any single SNP (**Supplementary Figure 4**). Meanwhile, results of the statistical power were 1.00 for both sexes.

## 4 Discussion

A number of previous observational studies have suggested a relationship between serum sex hormone levels and the risk of lower extremity varicose veins (14–16). However, observational studies are prone to biases such as reverse causality and unmeasured confounding (36). Randomized controlled trials may avoid some of these defects but are much too costly and time-consuming, and there may be no appropriate intervention to verify certain hypotheses in some cases (36). Hence, there is no randomized controlled study focused on the relationship between serum sex hormone levels and lower extremity varicose veins yet. Moreover, less attention has been paid to the association between serum SHBG and lower extremity varicose veins. As a result, it remained unclear whether there existing associations between the serum levels of SHBG and sex hormones and the risk of lower extremity varicose veins. In our study, the combination of the univariable MR, MVMR, and gender-stratified MR provided a novel solution.

We first explored the possible associations between serum SHBG and sex hormones levels and lower extremity varicose veins using univariable MR analysis. We found that increased serum SHBG levels were genetically associated with an increased risk of lower extremity varicose veins (OR=1.39; 95% CI: 1.13–1.70; P=1.58×10^-3^). Then, considering the interrelated nature between SHBG, testosterone and estradiol, we further performed an MVMR analysis to estimate the independent causal effect of serum SHBG levels on lower extremity varicose veins. Results showed that the causal effect of serum SHBG levels on lower extremity varicose veins risk was retained (OR=1.50; 95% CI:1.13-1.99; P=5.61×10^-3^), indicating that serum SHBG levels had an independent causal effect on lower extremity varicose veins risk. Lastly, we performed a gender-stratified MR analysis to investigate if there was a gender difference in the causal effect between serum SHBG levels and lower extremity varicose veins risk. Analysis showed that serum SHBG levels were associated with lower extremity varicose veins risk in both sexes. Notably, women’s risk for varicose veins increased to a greater extent than men’s when faced with increased serum SHBG levels. Specifically, for 1-standard deviation increase in serum SHBG levels, the risk of lower extremity varicose veins increases 51% and 26% in females and males, respectively.

By combining evidences from the univariable MR analysis, MVMR analysis and the gender-stratified MR analysis, we believe that, compared to individuals with normal SHBG levels, people with higher serum SHBG levels are more likely to suffer from lower extremity varicose veins. Notably, although serum SHBG levels were associated with lower extremity varicose veins risk in both sexes, the association seemed to be stronger in females. Besides, it has been widely proved that the SHBG level of middle-aged and elderly women is higher than that of men of the same age, and the data for SHBG and lower extremity varicose veins that we included were also from participants in this age group (37–39). Therefore, it can be further inferred that the stronger association between SHBG and lower extremity varicose veins in females may partly explain the fact that veins are more prevalent among females (3, 4). There are some reasons that might help to understand the causal relationship. Firstly, some studies have found that elevated serum SHBG levels are associated with a higher risk of cardiovascular disease (40), ischemic stroke, and heart failure in men (41) and a higher risk of venous thromboembolism in women (42). Besides, increased serum SHBG levels were associated with an increased risk of all-cause mortality in dysglycemic women (43). Particularly, elevated serum SHBG levels have also been found to be associated with an increased risk of aneurysmal subarachnoid hemorrhage risk only in women, not in men (44). These studies indicate that serum SHBG may be harmful to the vascular system, especially in women, with the specific mechanism remaining to be further studied though. Moreover, there is a positive relationship between high serum SHBG levels and increased risk of the frailty phenotype (45, 46). And some components of the frailty phenotype, such as low physical activity and slowness, have been shown to be associated with the risk of lower extremity varicose veins (1, 47–49).

Our findings showed a positive association of serum SHBG levels with the risk of lower extremity varicose veins in both sexes, especially in females, and extended the limited evidence concerning the role of serum SHBG levels in lower extremity varicose veins. Still, several limitations should be taken into account. First, while our sensitivity analyses incorporating the MR-Egger intercept test failed to find evidence of horizontal pleiotropy, it is still possible that vertical pleiotropy may be present (50). Second, while the causal relationship between serum SHBG levels and lower extremity varicose veins was revealed, the underlying mechanism is still equivocal and further research is needed. Finally, as this study was performed on participants of European descent, the results may not necessarily be generalized to other ethnic groups. Finally, since women’s SHBG levels vary before and after menopause, further exploration is needed to see if this causal association is different between postmenopausal and premenopausal women.

## 5 Conclusions

In summary, this is the first MR study to reveal the associations between serum SHBG levels and the risk of lower extremity varicose veins at gender-stratified level. We find that serum SHBG levels are positively related with lower extremity varicose veins risk in both sexes, especially in females. This may partly explain the higher prevalence of varicose veins among females.

## Data availability statement

The original contributions presented in the study are included in the article/**Supplementary Material**. Further inquiries can be directed to the corresponding authors.

## Ethics statement

Ethical approval was not required for the study involving humans in accordance with the local legislation and institutional requirements. Written informed consent to participate in this study was not required from the participants or the participants’ legal guardians/next of kin in accordance with the national legislation and the institutional requirements.

## Author contributions

QF, YM, SX, and CC designed the study. QF, YM, and ZN analyzed and interpreted the data. QF and YM were major contributors in writing the manuscript. SX and CC reviewed and edited the manuscript. All authors contributed to the article and approved the submitted version.
