# Supplementary figures and images for: Sex hormone-binding globulin exerts sex-related causal effects on lower extremity varicose veins: evidence from gender-stratified Mendelian randomization
Source: Front Endocrinol (Lausanne). 2023 Dec 11;14:1230955. doi: 10.3389/fendo.2023.1230955 (PMC10752419; doi:10.3389/fendo.2023.1230955)

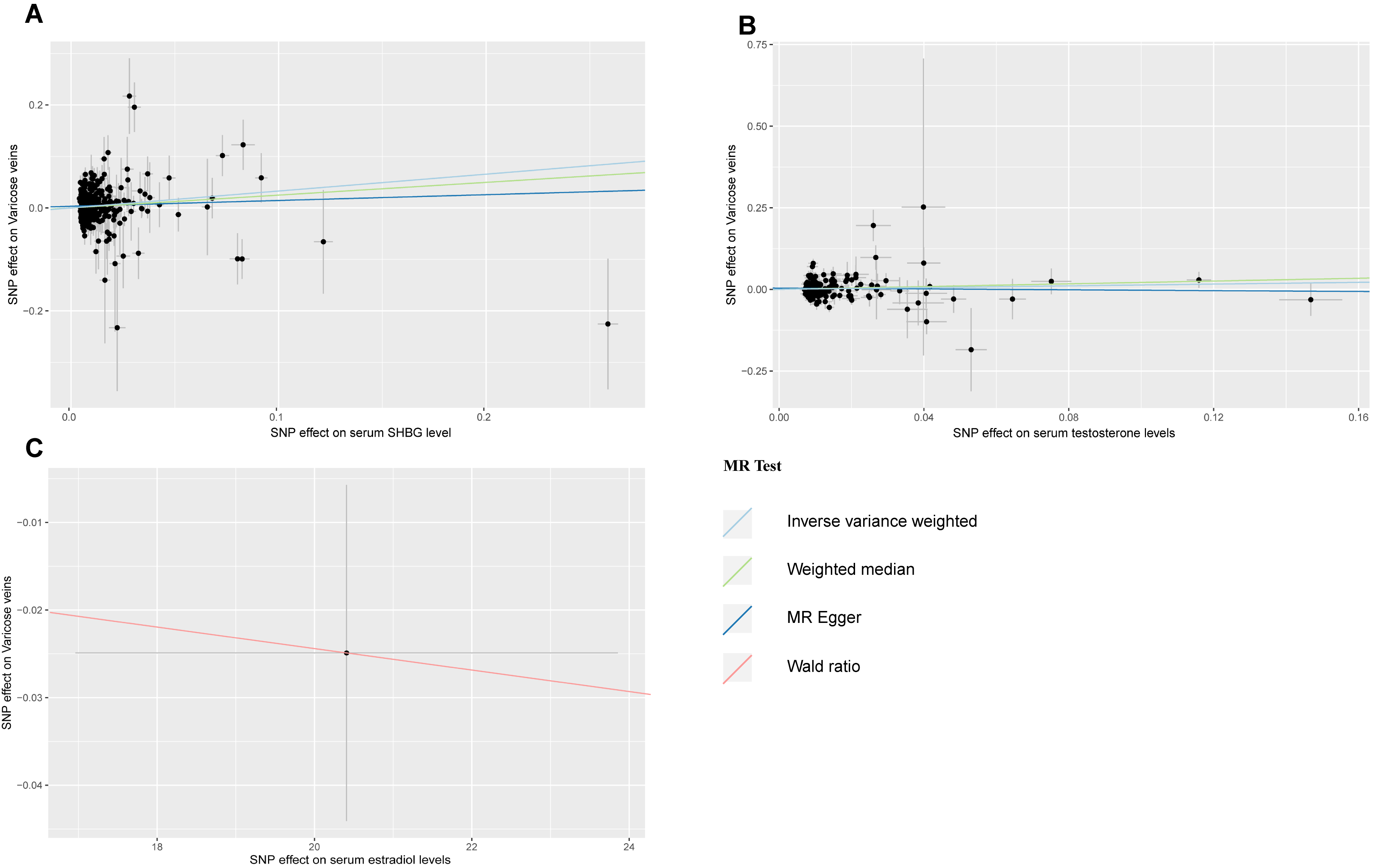

Supplement: Supplementary file 2 [file Image_1.tif]

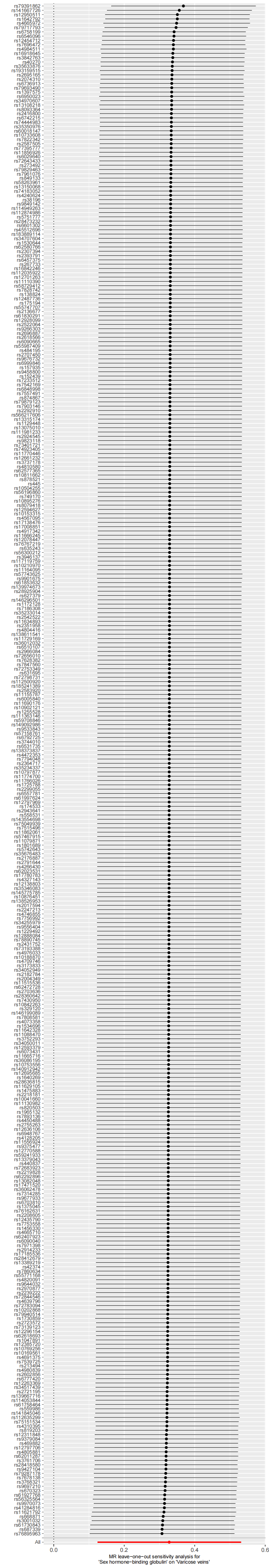

Supplement: Supplementary file 3 [file Image_2.tif]

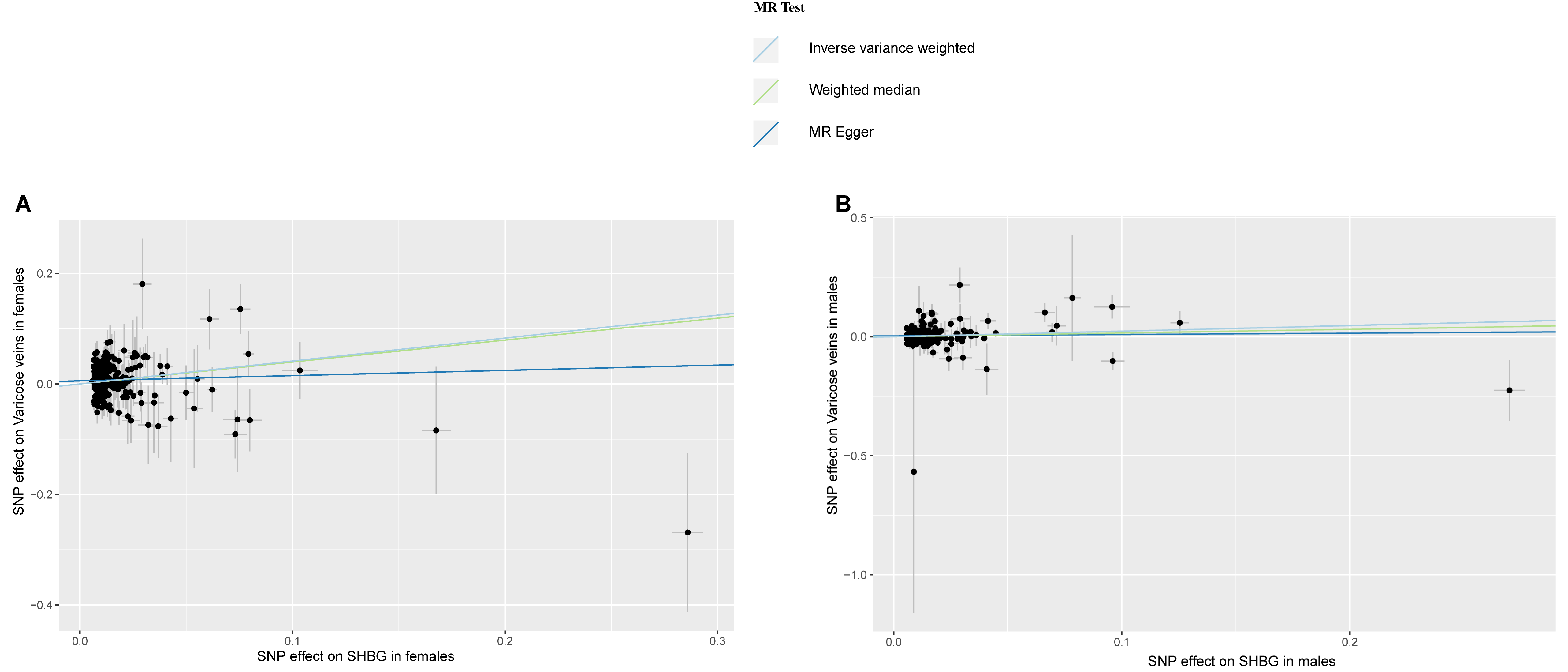

Supplement: Supplementary file 4 [file Image_3.tif]

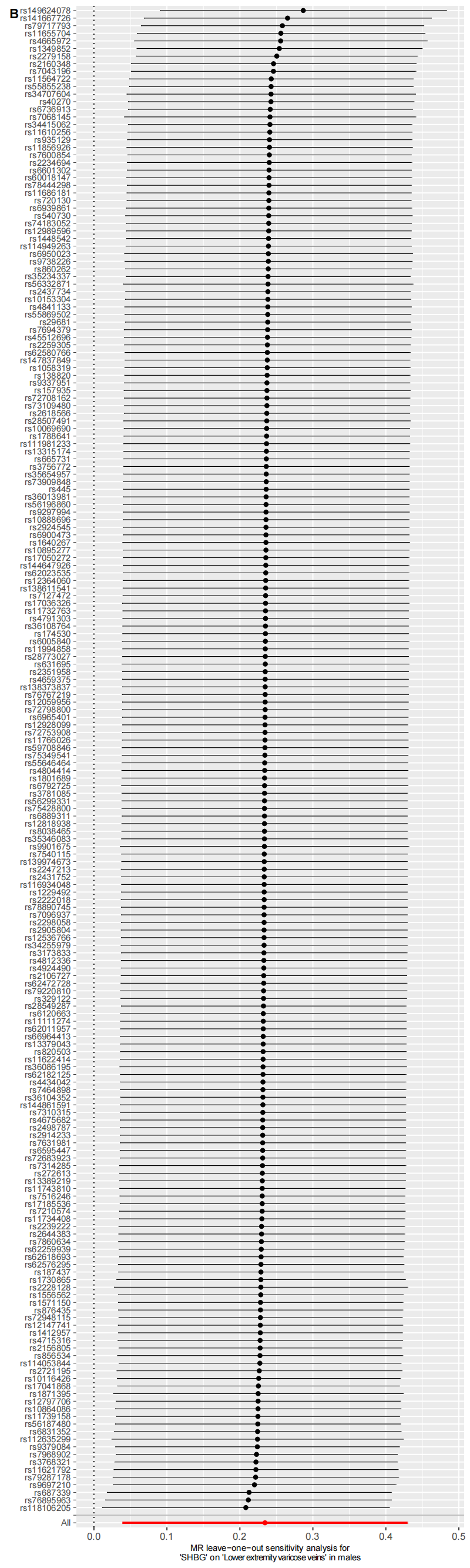

Supplement: Supplementary file 5 [file Image_4.tif]
